# The origin, connectivity, and individual specialization of island wolves after deer extirpation

**DOI:** 10.1002/ece3.11266

**Published:** 2024-04-16

**Authors:** Charlotte E. Eriksson, Gretchen H. Roffler, Jennifer M. Allen, Alex Lewis, Taal Levi

**Affiliations:** ^1^ Department of Fisheries, Wildlife, and Conservation Sciences Oregon State University Corvallis Oregon USA; ^2^ Alaska Department of Fish and Game Division of Wildlife Conservation Douglas Alaska USA

**Keywords:** *Canis lupus*, diet specialization, DNA metabarcoding, marine subsidies, noninvasive genetics, SNP genotyping

## Abstract

Wolves are assumed to be ungulate obligates, however, a recently described pack on Pleasant Island, Alaska USA, is persisting on sea otters and other marine resources without ungulate prey, violating this long‐held assumption. We address questions about these wolves regarding their origin and fate, degree of isolation, risk of inbreeding depression, and diet specialization by individual and sex. We applied DNA metabarcoding and genotyping by amplicon sequencing using 957 scats collected from 2016 to 2022, and reduced representation sequencing of tissue samples to establish a detailed understanding of Pleasant Island wolf ecology and compare them with adjacent mainland wolves. Dietary overlap was higher among individual wolves on Pleasant Island (Pianka's index mean 0.95 ± 0.03) compared to mainland wolves (0.70 ± 0.21). The individual diets of island wolves were dominated by sea otter, ranging from 40.6% to 63.2% weighted percent of occurrence (wPOO) (mean 55.5 ± 8.7). In contrast, individual mainland wolves primarily fed on ungulates (42.2 ± 21.3) or voles during a population outbreak (31.2 ± 23.2). We traced the origin of the Pleasant Island pack to a mainland pair that colonized around 2013 and produced several litters. After this breeding pair was killed, their female offspring and an immigrant male became the new breeders in 2019. We detected 20 individuals of which 8 (40%) were trapped and killed while two died of natural causes during the 6‐year study. Except for the new breeding male, the pedigree analysis and genotype results showed no additional movement to or from the island, indicating limited dispersal but no evidence of inbreeding. Our findings suggest wolves exhibit more flexible foraging behavior than previously believed, and hunting strategies can substantially differ between individuals within or between packs. Nevertheless, anthropogenic and natural mortality combined with limited connectivity to the mainland may inhibit the continued persistence of Pleasant Island wolves.

## Graphical abstract

We applied DNA metabarcoding and SNP genotyping to quantify variation in marine foraging behavior within wolf packs, among sexes, and individuals using 957 scats collected from an island and the adjacent mainland from 2016 to 2022. Island wolves consistently consumed sea otters while mainland wolves fed mainly on ungulates and voles with more variation annually, among sexes and individuals, indicating both individual variation in the diet of a cooperatively hunting predator and varying hunting strategies among individuals within the same pack. We also found limited dispersal to and from the island with only one unrelated wolf coming from the mainland during the 6‐year study period. Our findings suggest wolves are more flexible in their foraging strategies than is commonly appreciated and that the recolonization of sea otters can allow wolves to persist on predominantly non‐ungulate, marine resources. Nevertheless, anthropogenic and natural mortality, and a lack of movement to and from the island may inhibit the continued persistence of the Pleasant Island wolves.

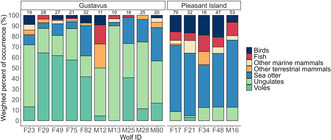

## 1 INTRODUCTION

The gray wolf (*Canis lupus*) is considered an ungulate specialist throughout its distribution and studies have consistently established a strong positive association between ungulate abundance and wolf density (Fuller et al., 2003; Messier, 1985, 1994; Serrouya et al., 2017). Wolves also demonstrate dietary flexibility through opportunistic consumption of alternative non‐ungulate species (Newsome et al., 2016). In coastal bioregions where ungulates are less abundant, wolves supplement their diet with allochthonous resources from marine environments, including salmon (*Oncorhynchus* spp.), and marine mammals (Adams et al., 2008; Darimont et al., 2009; Roffler et al., 2021; Watts et al., 2010). Nevertheless, even coastal and archipelagic wolves are thought to primarily feed on ungulates (Darimont et al., 2004; Massey et al., 2021; Roffler et al., 2021).

The obligate nature of the wolf‐ungulate relationship is further supported by the failure of wolves to persist during the classic Coronation Island (73 km^2^) wolf introduction experiment in Southeast Alaska (Klein, 1995). Wolves were introduced to Coronation Island to study the effects on resident Sitka black‐tailed deer (*Odocoileus hemionus sitkensis*). The wolf population increased until the deer population became functionally extirpated, forcing the wolves to consume alternative prey until the wolf population declined to a single individual after resorting to cannibalism (Klein, 1995). No known immigration or emigration occurred despite being well within dispersal distance to other islands where deer density remained high. Slow rates of immigration can influence the population dynamics of wolves on islands because immigration must be high relative to death rates to maintain isolated populations (Lomolino, 1986) exemplified by the near demise of wolves on Isle Royale (Robinson et al., 2019). Island populations may therefore be especially vulnerable to mortalities that increase the death rate relative to the immigration or birth rate, including human‐caused mortality.

In a striking exception to the assumed obligate relationship between wolves and ungulates, Roffler et al. (2023) described a wolf pack in Southeast Alaska that feeds almost entirely on marine resources. In 2013, wolves colonized Pleasant Island (49 km^2^) near Glacier Bay. Initially, the wolves fed primarily on deer, but deer declined rapidly until there were no detections from pellet surveys in either 2018 or 2021 for the first time in 30 years of monitoring (Roffler et al., 2023). As deer abundance declined, wolves prey‐switched to sea otters (*Enhydra lutris*), which had become abundant due to legal protection and reintroductions initiated in 1965 (Roffler et al., 2023; Tinker et al., 2019). Roffler et al. (2023) suggested that sea otters and deer were engaged in wolf‐mediated apparent competition (Holt, 1977) such that the availability of sea otters facilitated the near extirpation of deer by allowing wolves to persist on the island. Given their dependence on ungulates, wolves on Pleasant Island could have been expected to disperse to the nearby mainland (1.5 km) or have reduced birth rates, increased mortality, and a declining population density when deer became rare. Surprisingly, no movement of wolves has been detected between the island and the mainland based on genotyped scats and GPS data (Roffler et al., 2023). Thus, the wolves on Pleasant Island appear to be a closed population isolated from other nearby packs, which raises the potential risk for inbreeding. Further, the Pleasant Island pack is hunted and trapped during the annual harvest season, and it remains unknown how this impacts the viability of the wolves without compensatory immigration.

Thus, the Pleasant Island wolves appear to be persisting under these unusual conditions (1) without ungulate prey on a mostly marine diet, (2) in relative isolation from other wolf packs, and (3) with human‐caused mortality without frequent recolonization. Additional questions about how wolves can persist under these circumstances hold the potential to reveal novel insights into wolf ecology. Principal among these are: what are the origins of and how isolated is this pack from the mainland? Does isolation from the mainland pose a risk of inbreeding depression? How pervasive is this diet specialized on sea otters among individual wolves and/or sexes within the pack? Is this foraging behavior widespread? Finally, and most critically, are these wolves able to persist long‐term on their marine‐derived diet despite continued anthropogenic pressures?

Here, we use molecular methods to establish a detailed understanding of the ecology of wolves on Pleasant Island and compare them with the adjacent Gustavus Forelands mainland wolves that consume a more typical diet dominated by large mammals including moose (*Alces alces*), deer, black bears (*Ursus americanus*), and beavers (*Castor canadensis*) while also having access to sea otters and other marine prey such as salmon (Roffler et al., 2023). The island and mainland wolves represent two wolf packs with home ranges previously determined with GPS data in Roffler et al. (2023). We applied DNA metabarcoding and genotyping by amplicon sequencing to quantify variation in marine foraging behavior within the packs, sex, and individuals using wolf scats collected from 2016 to 2022. We also used tissue samples from trapped or captured wolves to quantify relatedness and gene flow between island and mainland wolves using reduced representation sequencing. Finally, we use this information to provide a timeline of the origin and fate of the Pleasant Island wolves.

## 2 MATERIALS AND METHODS

### 2.1 Study area

**FIGURE 1:**
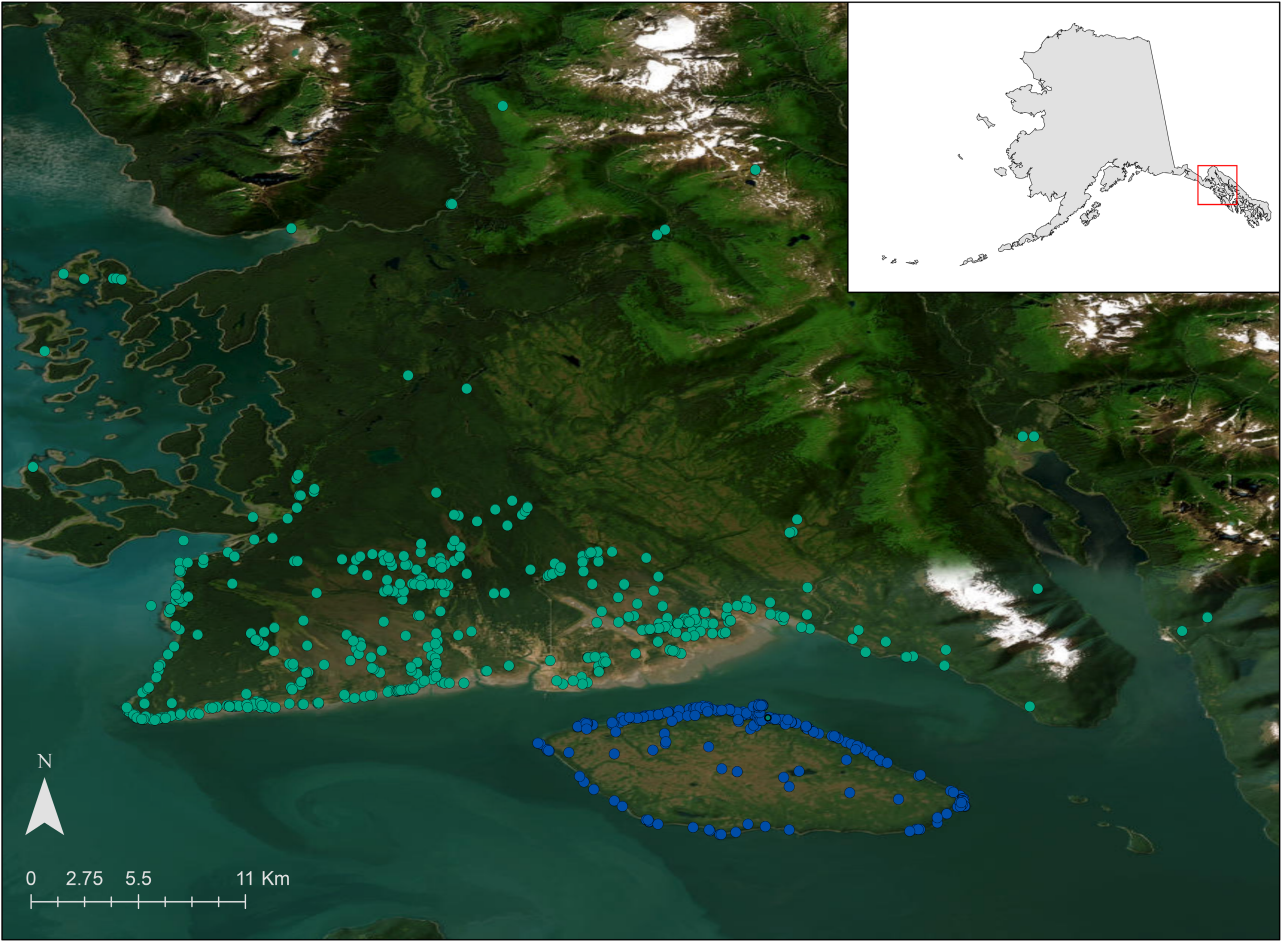
Map of study areas in Southeast Alaska. Points represent scat locations on Gustavus (green) and Pleasant Island (blue) from 2016 to 2022.

Gustavus and Pleasant Island are located near Glacier Bay, Southeast Alaska (135°49′33.8″ W, 58°25′35.1″ N) (Figure 1). The vegetation is characterized by temperate rainforest dominated by western hemlock (*Tsuga heterophylla*), Sitka spruce (*Picea sitchensis*), lodgepole pine (*Pinus contorta*), and willow (*Salix* spp.). The maritime climate is cool and wet with an average precipitation of 1400 mm per year and average temperature ranges from −7°C to 17°C (Western Regional Climate Center, 2016).

### 2.2 Sample collection and DNA extraction

We used the scat dataset from 2016 to 2020 that was originally reported in Roffler et al. (2023) and continued sample collection in 2021 and 2022. Scats were georeferenced and stored frozen in individual zip lock bags until processed in the laboratory. Tissue samples were obtained from trapped (*n* = 28) or captured (*n* = 12) wolves from Pleasant Island, two nearby mainland packs near Gustavus, and from more distant wolves near Haines, Juneau and on Kuiu, Kupreanof, and Prince of Wales Islands. See Roffler et al. (2023) for detailed scat collection and wolf capturing methods. We additionally collected the jaw from the remains of a dead wolf found during fieldwork on Pleasant Island and sampled a tooth.

We used a razor blade and tweezers to cut three small subsamples from each scat and placed them in a 2 mL Eppendorf tube. Tools were soaked in 50% bleach, rinsed with deionized water, and placed under UV light for 2 min between samples. The tissue samples were extracted using the DNeasy Blood and Tissue Kit (Qiagen) following the manufacturer's protocol. The scat samples were extracted with the same kit but using the following modifications: 550 μL buffer ATL, 50 μL Proteinase *K*, and a 4 h incubation. One negative control was included per extraction batch to monitor for contamination. We extracted DNA from the tooth sample following the protocol in Dabney and Meyer (2019).

We used DNA metabarcoding to identify the predator and vertebrate prey species present in the scats by amplifying ~100 bp of the ribosomal mitochondrial 12S gene region using slightly modified universal vertebrate primers 12SV5F (TTAGATACCCCACTATGC) and 12SV5R (YAGAACAGGCTCCTCTAG) (adapted from Riaz et al. (2011)). We performed three PCR replicates per sample and included three no‐template controls per 96‐well plate to monitor for cross‐contamination. Each PCR reaction was amplified with identical unique 8 bp tags on the 5′ end of the forward and reverse primers for assignment of sequence reads to the correct sample and to prevent tag jumping (Schnell et al., 2015). PCR plates were prepared in HEPA‐filtered and UV‐irradiated PCR cabinets within a pre‐PCR laboratory in 20 μL reactions with 10 μL Amplitaq Gold Master mix (ThermoFisher Scientific), 3 μL water, 5 μL of each primer (final concentration 200 nM), and 2 μL DNA template. Cycling conditions were 95°C initial denaturation for 10 min, followed by 35 cycles of 95°C for 30 s, 58°C for 30 s, 72°C for 60 s, and a final extension at 72°C for 7 min. We quantified the PCR products using a fluorescence microplate reader with the AccuBlue dsDNA High Sensitivity Quantitation Kit (Biotium) and normalized each sample accordingly to achieve even sequencing depth per sample. We pooled 384 samples per library and used NEBNext Ultra II Library Prep Kit (New England Biolabs) to adapt the library pools into Illumina sequencing libraries following the manufacturer's instructions (Illumina Inc). The libraries were purified using PCRClean DX (Aline Biosciences), quantified using AccuGreen Broad Range dsDNA Quantitation Kit (Biotium), and normalized before sent for 150 bp paired‐end sequencing on an Illumina HiSeq 3000 or Nextseq 2000 at the Center for Quantitative Life Sciences (CQLS), Oregon State University.

We used *PEAR* (Zhang et al., 2013) to pair the raw sequence reads and then demultiplexed the paired reads using the unique 8 bp‐index sequences with a custom shell script. Unique reads from each sample replicate were counted and taxonomically assigned using *BLAST* (www.ncbi.nlm.nih.gov/blast), against all 12S vertebrate sequences in Genbank supplemented with a custom 12S library created for vertebrates not present in Genbank (Eriksson et al., 2019). We assigned taxa with 100% match to species level if present in at least two out of the three replicates. We assigned the taxa to genus or family level if several locally occurring species matched the sequence to 100% or if the match was <99%. We removed sequences that made up <0.5% of the total number of sequences for a sample. We grouped prey species into diet item categories and calculated the weighted percent of occurrence (wPOO) of each prey category (Deagle et al., 2019).

Individual diet variation was quantified based on wolves with a sample size of at least 10 scats based on the recommendations by Prugh et al. (2008). We calculated the Bray–Curtis dissimilarity index between each pair of wolves and visualized the dietary dissimilarities using nonmetric multidimensional scaling (NMDS) in the R package vegan (Oksanen et al., 2022). We conducted pairwise permanova using “pairwise. adonis” in the funfuns package (Trachsel, 2021) to test for differences in diet composition between individual wolves while correcting for multiple comparisons using the false‐discovery rate method. Dietary overlap was further evaluated using Pianka's overlap index in the r package “spaa” (Pianka, 1973; Zhang et al., 2016) where a value of 0 indicates no overlap and 1 represents total overlap.

### 2.3 SNP genotyping

We used the SNP panel designed for individual identification of wolves in Roffler et al. (2023). This panel also contains three SNPs with wolf versus coyote‐specific alleles and a sex‐specific primer pair targeting 80 bp of the Y chromosome to determine the sex of individual wolves. The SNP panel had 38 loci, which is a sufficient number of loci for individual identification (Hayward et al., 2022). SNP primer sequences are available in the Supplemental Data S1. We estimated the probability of identity in GenAlEx 6.5 (Peakall & Smouse, 2012) to determine the power of our SNP panel to distinguish between individual wolves (PID = 5.3 × 10^−12^ and PIDsibs 1.6 × 10^−6^).

Our genotyping protocol followed the methods described in Eriksson et al. (2020). Briefly, genotyping involved first amplifying all SNP primers with overhanging Illumina adapters in a multiplex PCR, the PCR products were then purified and used as template in a second PCR where unique index barcodes were added to each sample. The products from the second PCR were then quantified, normalized, and pooled. The pools were purified, quantified, and normalized before submitted for sequencing on an Illumina HiSeq 3000 or Nextseq 2000 at CQLS, Oregon State University. Each sample was genotyped in triplicate and each 96‐well plate contained three no‐template controls to monitor for cross‐contamination. All PCRs were performed in HEPA‐filtered and UV‐irradiated PCR cabinets. Wolf tissue extracts were prepared on separate PCR plates using a different PCR cabinet and pipettes to prevent cross‐contamination with the lower quality scat samples.

The raw sequences were demultiplexed based on their unique index combinations using *bcl2fastq* (Illumina Inc). Genotypes were assigned based on read counts for each allele using *perl* scripts by Campbell et al. (2015). We required a heterozygous genotype to be observed in two out of three replicates and all three replicates to call a homozygous genotype. Samples that failed to produce a genotype in >20% of loci were removed. We performed a fragment length analysis of the mitochondrial control region on an AB3730 capillary sequencer to validate the accuracy of our three wolf versus coyote‐specific SNPs, as described in (Roffler et al., 2021).

### 2.4 Reduced representation sequencing

We submitted the 40 tissue extracts described above for reduced representation sequencing at CQLS. Briefly, the DNA extracts were normalized to 20 ng/μL and digested with PstI (CTGCAG) and MspI (CCGG). Adapters with unique barcodes were ligated followed by pooling and purification using QIAquick PCR Purification Kit (Qiagen). The ligated samples were PCR‐amplified for 15 cycles consisting of 98°C (10 min), 68°C (30 s), 72°C (30 s), 72°C (5 min) and then sequenced on a NextSeq 2000 P2 150 bp paired‐end run.

We demultiplexed the raw reads using the process_radtags module with default parameters in *STACKS* (Catchen et al., 2013). The resulting sequences were mapped to the dog genome (GCA_000002285.2_CanFam3.1) using bwa (Li & Durbin, 2009). We used *SAMtools* (Li et al., 2009) to sort the alignments and convert to BAM format. We created a SNP catalog using the “gstacks” module in *STACKS* with default parameters (‐‐model marukilow and ‐‐var‐alpha 0.05) and then ran the “populations” module with the “‐‐write_single_snp” flag. We used *PLINK* (Purcell et al., 2007) to produce a heavily filtered dataset of between 300 and 700 SNPs for pedigree analysis as recommended for the r package Sequoia (Huisman, 2017). Our filtering parameters were as follows: ‐‐biallelic‐only ‐‐snps‐only ‐‐geno 0.1 ‐‐maf 0.3 ‐‐hwe 0.001. We further excluded loci in statistical linkage disequilibrium using “indep‐pairwise 50 5 0.5” and removed sex‐linked loci. Finally, we excluded samples with >30% missingness.

The estimation of allele frequencies and relatedness from the same samples assumes the dataset consists of unrelated and non‐inbred individuals. Since most of our sampled wolves likely originated from a few adjacent packs, we expected a large proportion of closely related individuals in our dataset. We therefore estimated relatedness using software “*EMIBD9*”, which iteratively updates the estimated allele frequencies and identical by descent (IBD) coefficients until convergence (Wang, 2022). We then reconstructed pedigrees using Sequoia with a relaxed error rate (Err = 0.01) following vonHoldt et al. (2020). Finally, we cross‐referenced our *EMIBD9* relatedness results with the Sequoia output to establish a consensus pedigree.

We used *STRUCTURE* (Pritchard et al., 2000) to estimate number of putative groups (*K*), i.e. wolf packs, and estimated the probability (*q*‐ or ancestral value) that an individual wolf descended from Pleasant Island or the mainland. A dataset including multiple close family members can lead to an overestimation of *K* leading to the identification of family lineages rather than larger scale population structure (Pritchard et al., 2000). *STRUCTURE* has therefore been used previously to assign pack membership of individual wolves (Bassing et al., 2020; Stansbury et al., 2016). We ran a general admixture model with correlated allele frequencies using a burn‐in of 10,000 and 50,000 MCMC repetitions for *K* = 1–5 and 10 iterations. We used *STRUCTURE HARVESTER* (Earl & vonHoldt, 2012) to determine the optimal *K* and then required *q*‐values of >0.7 to assign an individual to a pack based on Stansbury et al. (2016). We ran the same model using three datasets: (1) the tissue samples and the 653‐SNP dataset, (2) the tissue samples with the small 35‐SNP panel, (3) all unique genotypes (tissue and scat samples) with the small SNP panel. The coyote‐wolf specific loci (*n* = 3) and SRY locus were removed since only polymorphic markers were applicable for this analysis, resulting in 35 loci remaining in the small SNP panel.

## 3 RESULTS

We collected and sequenced 1243 putative wolf scats, resulting in 183,475,451 raw sequencing reads and 170,275.5 reads on average per sample. A total of 1198 samples were amplified successfully and passed filtering (96%). After excluding non‐wolf scats (*n* = 134), and scats that contained no other vertebrate DNA aside from wolf (*n* = 107), 957 samples remained for pack‐level diet analysis. Of these, 649 had been previously analyzed in Roffler et al. (2023). We added 308 new scat samples from 2021 to 2022 (Table S1). We attempted to genotype 1001 canid scats in total, which included all Pleasant Island scats (including samples that failed metabarcoding) and the majority of the Gustavus scats (including coyote samples). We did not attempt to genotype the Gustavus scats that failed metabarcoding and chose a random subset of the 2021–2022 scats due to the large sample size relative to Pleasant Island. A total of 723 canid scats (including 62 coyote scats) were genotyped successfully resulting in a genotyping success rate of 72%. All tissue samples were genotyped successfully. Based on scat and tissue genotypes, we identified 20 individual wolves (12 females and eight males) from Pleasant Island and 51 wolves (22 females and 29 males) from Gustavus during our study period. For the sex and individual‐level diet analysis, we removed genotyped wolf scats that contained no vertebrate prey DNA (*n* = 76) resulting in 585 remaining samples.

### 3.1 Wolf diets

**FIGURE 2:**
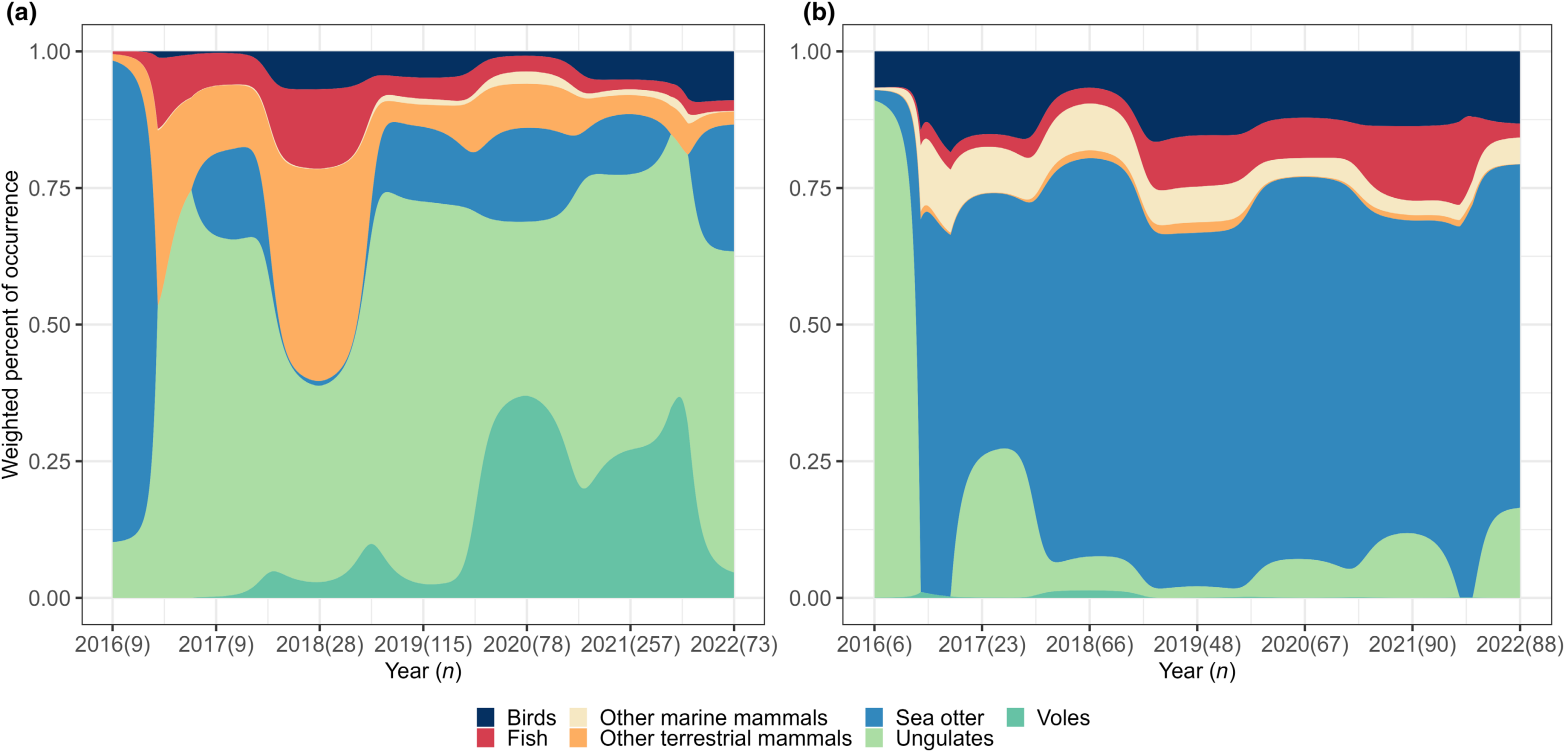
Annual changes in dietary composition of the wolf pack on Gustavus (a) and Pleasant Island (b) based on DNA metabarcoding of 957 scats collected from 2016 to 2022 (Alaska, USA). Prey species were grouped into taxonomic categories and presented as weighted percent of occurrence. Sample sizes per year are shown within parentheses.

The general pack‐level diet patterns from previous years (2016–2020) continued in 2021–2022 (Figure 2; Figure S1). In total, Pleasant Island scats (*n* = 388) were dominated by sea otter (63.2% wPOO), while wolves on the mainland (*n* = 569) fed mainly on ungulates (51.8%), voles (19.5%), and sea otter to a lesser extent (14.3%). A variety of seabirds made up the second most common prey category consumed by the island wolves (12.3%) (Figure S1; Table S2). These patterns remained consistent throughout the years for the Pleasant Island wolves, while annual diet composition was more variable for the Gustavus pack (Figure 2). The diet composition for both packs in 2016 was likely influenced by the small sample size (*n* < 10) and may not be representative. Overall, the proportion of marine versus terrestrial prey items varied between the two packs with 77.5% of prey items in mainland scats coming from terrestrial sources and 22.5% were marine. In contrast, the prey items consumed by the island wolves were 83.0% marine and 17.0% terrestrial (for species‐level diet see Table S2).

**FIGURE 3:**
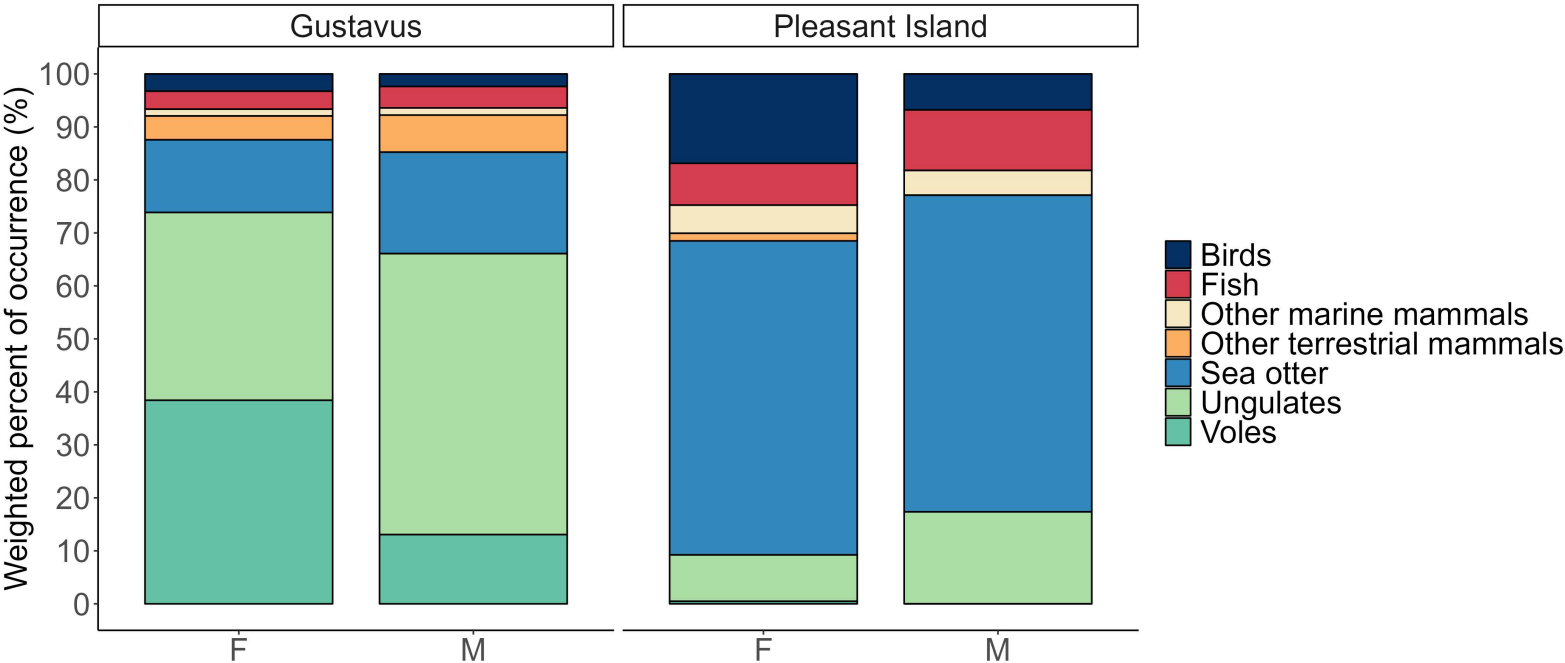
Weighted percent of occurrence of prey categories in male (M) and female (F) wolf diets on Gustavus and Pleasant Island, Alaska, USA from 2016 to 2022.

The diets of both male (*n* = 59 scats) and female wolves (*n* = 210 scats) on Pleasant Island were heavily dominated by sea otter followed by seabirds and fish (Figure 3). In contrast, male wolves on Gustavus (*n* = 148 scats) mainly preyed on moose and sea otters while females (*n* = 169 scats) primarily consumed voles (mainly *Microtus* spp.), moose, and sea otters (Figure 3).

**FIGURE 4:**
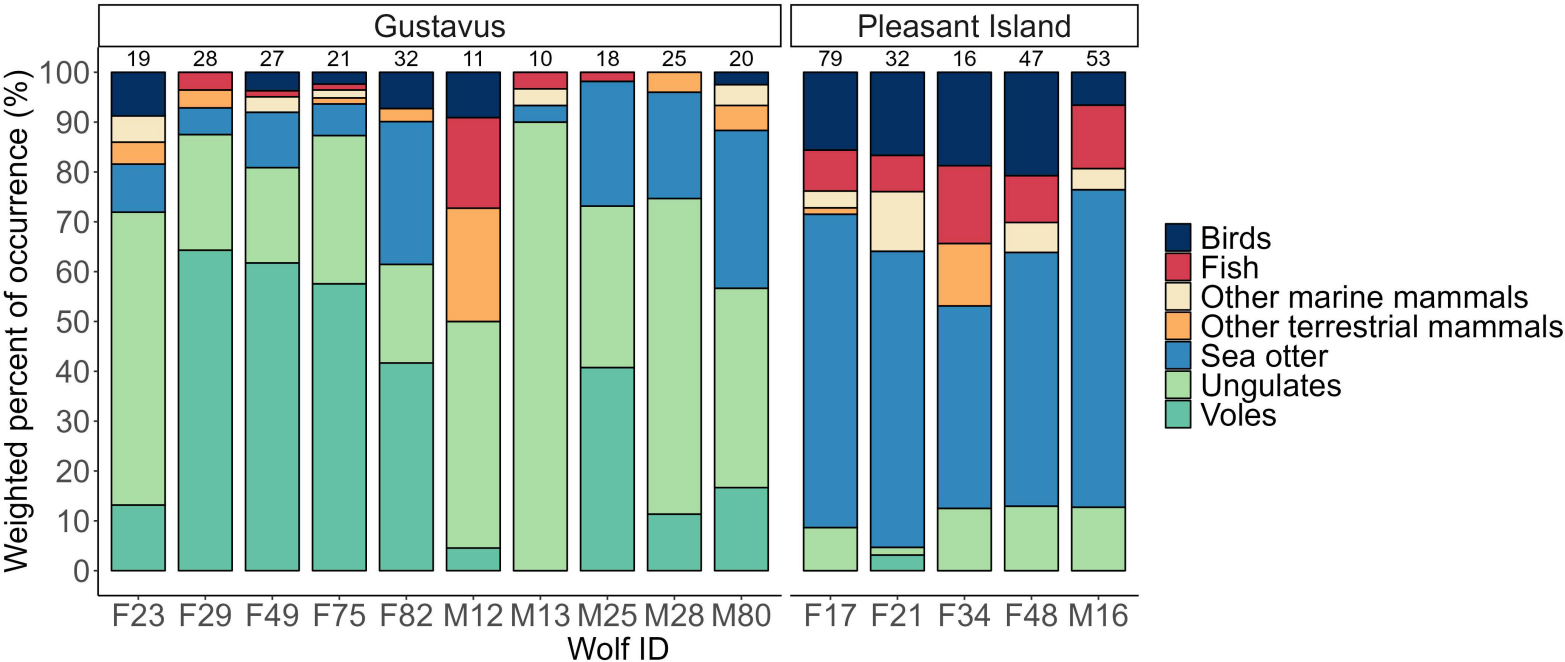
Weighted percent of occurrence of prey categories in individual wolf diets on Gustavus and Pleasant Island (Alaska, USA) based on DNA metabarcoding of scats collected from 2016 to 2022. The number on top of each bar represents the number of scats per wolf. ‘F’ = Female and ‘M’ = Male.

The number of scats per individual wolf ranged from 1 to 79 including a total of 15 wolves (Gustavus = 10 and Pleasant Island = 5) with sample sizes of ≥10 (mean = 29.2 ± 18.2 SD). Sea otter dominated the diet of all Pleasant Island individuals (mean 55.5% ± 9.7 wPOO, Figure 4). Individual diets of the Gustavus pack were more variable (Figure 4). The female wolves on Gustavus fed mainly on voles except for the breeding female, F23, that consumed mostly ungulates, similar to the male wolf diets. Sea otter consumption by the Gustavus wolves ranged from 0 to 31.7% (mean 14.2% ± 11.4 wPOO, Figure 4). The high use of voles seems to be opportunistic and tied to a large vole population outbreak in 2020–2021 (G. Roffler, personal communication, May 2022), also noticeable in the overall annual diet of the Gustavus wolves (Figure 2). In comparison, sea otter consumption did not fluctuate and was consistent throughout the years indicating a steady resource availability for the island wolves (Figure 2).

**FIGURE 5:**
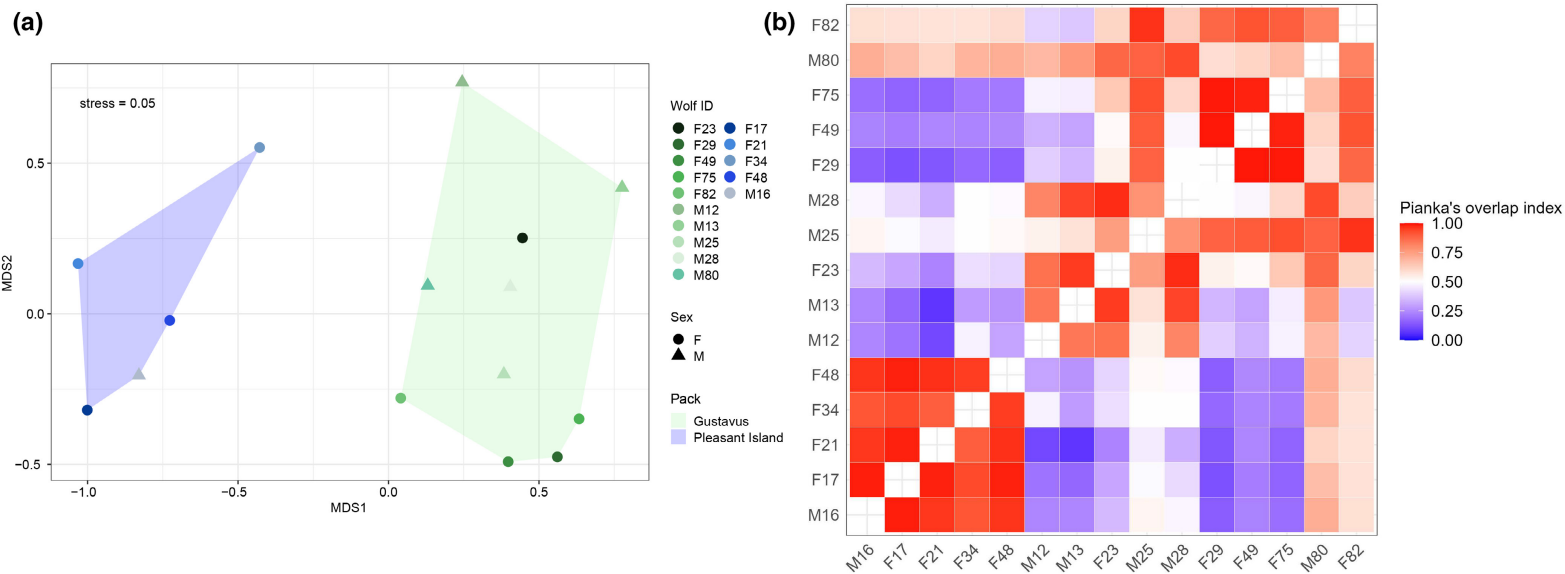
(a) Dietary niche partitioning among packs and individual wolves based on nonmetric multidimensional scaling (NMDS) according to the Bray–Curtis Dissimilarity index. Points are represented as the pooled diet per individual. Points in closer proximity to one another indicate more similar diets; polygons are convex hulls around all individuals from each pack. (b) Pianka's index of dietary niche overlap among individual wolves. 0 = no dietary overlap, 1 = complete dietary overlap. F48, F34, F21, F17, and M16 were from Pleasant Island and the remaining wolves were from Gustavus.

Nearly all of the pairwise comparisons (96%) between island and mainland wolves were significantly different (Table S3). In contrast, diet composition did not vary among any of the island wolves, while 51% of the pairwise contrasts among the Gustavus wolves were significantly different. These results were also evident by NMDS (Figure 5a) and further corroborated by Pianka's overlap index where pairwise comparisons of dietary niche overlap between individual wolves was high for Pleasant Island wolves (Pianka's index mean 0.95 ± 0.03) and lower for Gustavus wolves (mean 0.70 ± 0.21 SD) (Figure 5b).

### 3.2 Individual wolves and their origin

The total number of reads for the reduced‐representation sequencing of wolf tissues was 1,054,509,196 and ranged from 225,482 to 102,578,781 per sample (mean = 21,359,723.15 ± 19,400,546.65). After excluding three samples with <0.5% of the total raw reads and/or <50% aligned reads, 38 samples remained with a mean depth of coverage of 43.4 (range 12.0–85.6) and 507,506 SNPs. After filtering for minimum allele frequency, Hardy Weinberg equilibrium, and linkage disequilibrium, 653 highly informative SNPs remained. We assessed the samples for missingness and removed one additional sample with >30% missing loci.

**FIGURE 6:**
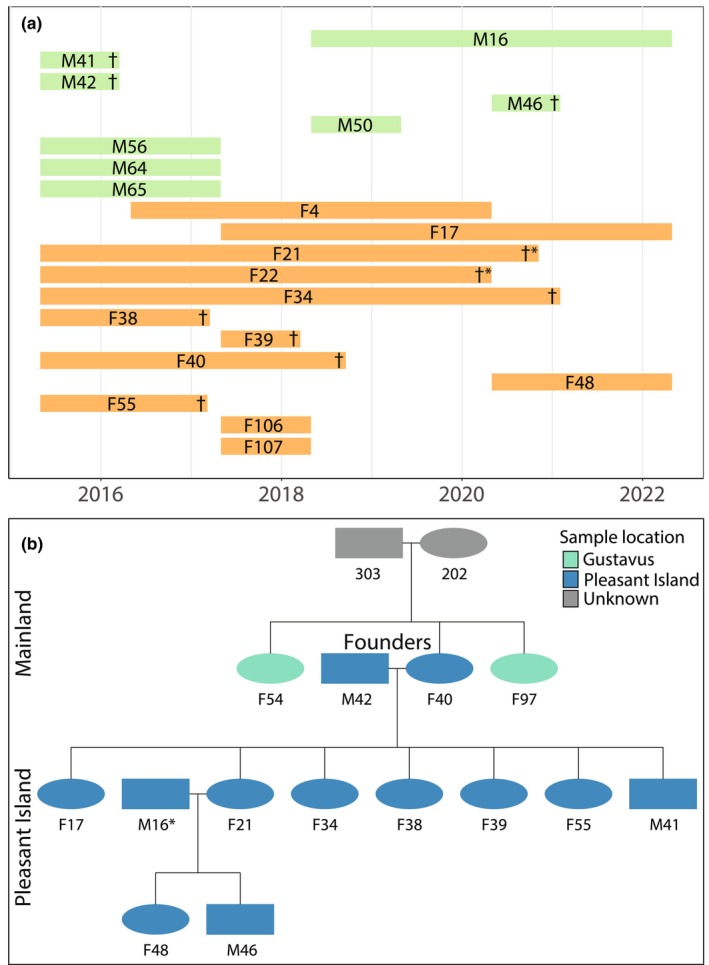
(a) Detection period and known fate of individual wolves on Pleasant Island per year from 2016 to 2022 based on genotypes from noninvasively collected scats, and tissues from trapped or captured wolves. Green and orange color represent males and females, respectively. † = human‐caused mortality, †* = natural causes of mortality. (b) Pedigree of the Pleasant Island pack based on 653 SNPs and tissue samples collected from killed or captured wolves. *We had no tissue sample from M16 but he could be assigned as the father because he was the only adult male present when F48 and M46 were born. Colors represent where samples were collected. “Unknown” refers to individuals that were not sampled.

**FIGURE 7:**
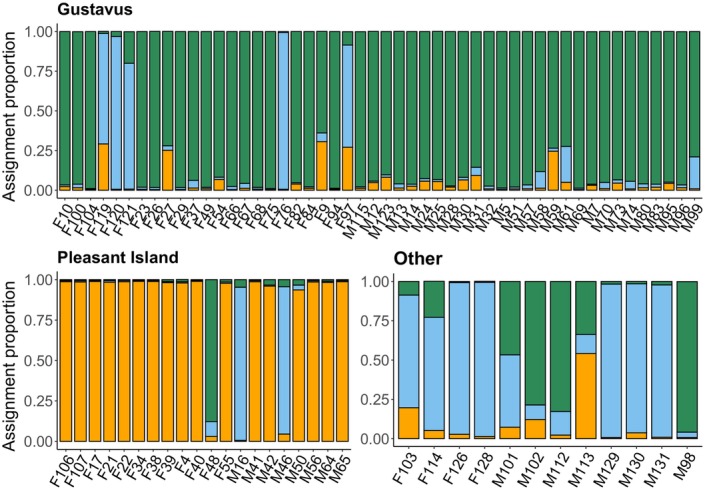
Proportion ancestry (pack) assignment for each unique wolf genotype based on scats and tissue samples genotyped with 35 SNPs. Each bar represents a unique genotype and the colors represent proportion ancestry to the different packs or territories. Most likely *K* value = 3. The regional samples that were not collected near Gustavus or on Pleasant Island have been grouped into “Other.”

Our pedigree analysis mainly corroborated the limited movement between Pleasant Island and the mainland as identified by previous scat genotyping and GPS data (Roffler et al., 2023). However, we now identify that wolves F40 and M42 came from the mainland and became the first breeding pair on Pleasant Island (Figure 6). F40 had two sisters (F97 and F54) that were trapped and killed on the Gustavus Forelands, suggesting that she came from the Gustavus pack originally. Based on the tissue samples, the breeding pair had at least seven pups (six females, one male) prior to both being trapped and killed, M42 in March 2016 and F40 in September 2018. Their daughter, F21 took over as the breeding female with a newcomer, M16, who likely came from the mainland to the island in 2019 based on scat detections (Figure 6a). Since M16 was never captured and is currently alive, we lack a tissue sample and therefore relatedness information from him. However, it is clear that he was the breeding male since he was the only adult male present at the time and he had no opposing homozygosity with the pups born in 2020. The genetic composition of his offspring F48 and M46 (Figure 7; Figure S2) showed clear divergence from the other wolves in the pack indicating that M16 originated from the mainland. Despite using a small number of loci (35) we were able to detect a strong signal of family structure clearly separating the Pleasant Island and mainland wolves (Figure 7), which was corroborated with the large SNP panel (Figure S2). These results indicate that after the arrival of M42 and F40, all the other remaining wolves, with the exception of M16, were born on the island. The pedigree analysis did not show evidence of inbreeding occurring (Figure 6b), and the inbreeding coefficient for the island wolves was also low (range 0.0006–0.0607 and average 0.012), however, we lack relatedness information from wolves sampled only from scats, and cannot say conclusively whether these individuals were inbred or not. Nevertheless, the timeline of detections suggests that all the wolves sampled only from scat DNA were the offspring of the founding pair except for potentially M50 who was first detected in 2018 (Figure 6a). M50 could be the offspring of the founding pair but not detected until then or could potentially be the result of inbreeding after the breeding male (M42) was killed in March 2016. The tenure of individual wolves on Pleasant Island varied between males and females according to our genotype detection histories (Figure 6a). Nearly all male wolves were only detected in 1 year except for M16 who was detected 4 years in a row (mean 1.4 ± 0.99 years). In contrast, five of the 12 females were detected annually for a period of 4–5 years (mean 2.7 ± 1.7 years) (Figure 6a). During this study, eight of the 20 genotyped individuals were trapped and killed. The second breeding female (F21) and another female (F22) died of natural causes and three wolves were alive at the end of this study (F48, M16, and F17). However, F17 was killed by a moose in summer 2023. The fate of the other five individuals remains unknown.

## 4 DISCUSSION

The gray wolf is one of the most well‐studied vertebrates in the world (Ripple et al., 2014), and knowledge of its ecology has provided iconic insights into the structure and persistence of small isolated populations, demonstrated through natural and intentional experiments such as Isle Royale and Coronation Island, respectively. Roffler et al. (2023) were the first to describe the Pleasant Island pack that forages predominantly on sea otters with minimal consumption of ungulates and suggested that the recovery of sea otters may allow wolves to expand to small island systems with associated cascading changes to terrestrial and near‐shore food webs. Here we expand upon this work by assessing whether this foraging strategy is consistent among males, females and individual wolves on the island and compare it to the adjacent mainland pack. We also explored the connectivity of the island wolves and the mainland using reduced representation sequencing and pedigree analysis.

We found a high dietary overlap among the island wolves and that use of marine resources, sea otters in particular, was consistent among sexes and individuals. In contrast, the mainland wolves exhibited more diet variation among both sexes and individuals. We documented an unusually large consumption of small prey species by the mainland wolves, in particular voles and birds, where the frequency of consumption varied by sex. Females on the Gustavus mainland consumed large amounts of voles (38.4% wPOO) in addition to ungulates (35.4%) while male wolves consumed primarily ungulates (53.0%) and considerably fewer voles (13.1%) (Figure 3). The reasons for the high rate of small prey consumption and discrepancy between sexes and individuals is unclear but could be due to social status or different foraging behaviors of males and females. Our finding that nonbreeding females capitalized on a vole population outbreak to such an extent that voles became the dominant prey item has to our knowledge not been described in wolf diet studies previously (Figure 4) and demonstrates that wolves are more flexible and opportunistic than is commonly appreciated. Such trends highlight the value of longitudinal studies where a snapshot study conducted for example in 2018 versus 2020 would have yielded very different results. In addition, our findings provide evidence of individual variation in the diet of a cooperatively hunting predator indicating varying hunting strategies between packs and among individual wolves within the same pack.

Previously, the Pleasant Island pack was thought to be functionally isolated because no genotyped individuals were detected on both the island and mainland (Roffler et al., 2023), however, using pedigree analysis, we documented two immigration events to the island. We found that the first breeding pair came from the mainland based on close relatedness with mainland wolves including full siblings in the Gustavus pack for the first breeding female, and that a second male dispersed to the island in 2019. However, we did not document any emigration from the island to the mainland. Thus, it remains unclear whether wolves avoid dispersing from the island due to social dynamics of wolf packs (Bassing et al., 2020) or whether the 1.5 km of water acts as a barrier. The latter seems unlikely given that wolves are capable swimmers and famous for their long‐distance dispersal abilities (Darimont & Paquet, 2002; Fuller, 1989; Stronen et al., 2014). We know that dispersal is possible, yet several females born on the island chose not to leave (Figure 6a). We also documented more females than males in the Pleasant Island pack (eight males and 12 females; 59 and 210 scats, respectively). It is unclear if there was a female‐biased sex ratio already at birth or whether juvenile males rapidly dispersed from the island or died without getting captured or detected through scats. Female wolves have a higher chance of becoming breeders in their natal pack compared to males (Ausband, 2022; Caniglia et al., 2014; Jędrzejewski et al., 2005), which may explain the lack of dispersal in adult females from the island. Wolves disperse more frequently when prey availability per capita is low (Morales‐González et al., 2021). It is possible that the marine subsidies are abundant enough such that wolves on Pleasant Island are not yet at local carrying capacity and the need to disperse is therefore alleviated. The continued harvest rate may further have maintained wolves below carrying capacity.

Overall, there was a declining trend in the number of wolves detected on the island over time (Figure 6a). Despite the overwhelmingly marine diet, the wolves on Pleasant Island were breeding nearly annually based on visual counts of wolf pups (Roffler et al., 2023), which suggests that they are able to meet their energetic requirements on a marine diet. It is possible the larger litters in the earlier years were enabled by the wolves predating a naïve population of deer that had little access to escape terrain or refugia. The number of wolves was reduced after the deer were depleted and wolves made the complete switch to sea otters. Average body mass from GPS‐collared wolves was somewhat higher for the mainland packs (28.7 ± 0.83 kg and 26.8 ± 0.72 kg for Gustavus and Pleasant Island, respectively) (G. Roffler, unpublished data) but the predominantly marine diet on Pleasant Island did not substantially affect body condition. A diet high in marine prey may provide the wolves with beneficial omega 3 fatty acids, typically limited in terrestrial prey (Twining et al., 2016), but also burden them with a high exposure to mercury (McGrew et al., 2014), a neurotoxin with known adverse effects to reproductive health in mammals (Wolfe et al., 1998). Nevertheless, the mechanisms driving the decline in wolf abundance on Pleasant Island could also be driven by factors unrelated to diet such as intraspecific conflict, winter severity or disease (Cubaynes et al., 2014), or by human‐caused mortality without frequent recolonization (Figure 6).

Although we were not able to investigate cause‐specific mortality for all individuals, we documented eight human‐caused deaths and two natural causes during the study period (Figure 6a). Two wolves were killed by trappers per biological year in 2016, 2017, 2018, and 2020 which constituted approximately a 23% harvest per year average. No wolves were killed in 2019 and 2021. Wolf populations can compensate for human‐caused mortality up to 29% of annual harvest via dispersal mechanisms including immigration and emigration (Adams et al., 2008). Ausband et al. (2015) found that recruitment declined after harvest both due to direct pup mortality but also indirect effects on group dynamics such as smaller group sizes. Pup survival rates have been shown to be lower in packs with fewer than 5 to 6‐members (Brainerd et al., 2008; Smith et al., 2010) likely due to important pup‐rearing support such as prey provisioning and protection provided by non‐breeders (Ruprecht et al., 2012). The Pleasant Island pack persisted despite a near annual harvest, including two breeders killed, with minimal compensatory immigration by maintaining fecundity on their primarily sea otter diet.

Our findings suggest that wolves are more flexible in their foraging behavior than previously believed, and hunting strategies can substantially differ between individuals within or between packs. Nevertheless, the future of the Pleasant Island pack remains uncertain due to the level of mortality whether human‐caused or from other sources such as mercury poisoning, without compensatory immigration. It is unclear if limited dispersal is widespread among island‐dwelling wolves or whether it is specific to those specializing on marine prey such that dispersal pressure is low due to abundant local resources. Future research is needed to understand how widespread the effects of the sea otter restoration success are on population size and demographic rates of coastal wolves. Additionally, community‐wide research is needed to understand how wolves decoupled from their typical ungulate prey may influence terrestrial and nearshore ecosystems. Our results suggest that small islands may be capable of sustaining new or additional packs in this region due to the recovering sea otter population, but that a combination of harvest pressure and lack of immigration may negate these opportunities. Pleasant Island provides a limited but exciting lens into these newly discovered dynamics and wolves here continue to remain despite these pressures.

## AUTHOR CONTRIBUTIONS

**Charlotte E. Eriksson:** Conceptualization (lead); formal analysis (lead); investigation (lead); methodology (lead); writing – original draft (lead); writing – review and editing (lead). **Gretchen H. Roffler:** Funding acquisition (lead); project administration (lead); resources (lead); writing – review and editing (supporting). **Jennifer M. Allen:** Data curation (lead); methodology (supporting); project administration (supporting); writing – review and editing (supporting). **Alex Lewis:** Data curation (supporting); investigation (supporting); writing – review and editing (supporting). **Taal Levi:** Conceptualization (supporting); investigation (supporting); supervision (lead); writing – review and editing (supporting).

## CONFLICT OF INTEREST STATEMENT

The authors declare no competing interests.

## Supporting information


Data S1.

Figure S1.
Table S1.
Table S2.
Table S3.

## Data Availability

Data are available at the Dryad Digital Repository https://doi.org/10.5061/dryad.hhmgqnkps.
