# Supplementary material for: The origin, connectivity, and individual specialization of island wolves after deer extirpation
Source: Ecol Evol. 2024 Apr 16;14(4):e11266. doi: 10.1002/ece3.11266 (PMC11021858; doi:10.1002/ece3.11266)
Supplement: Supplementary file 2 — Figure S1. Table S1. Table S2. Table S3. [file ECE3-14-e11266-s001.pdf]

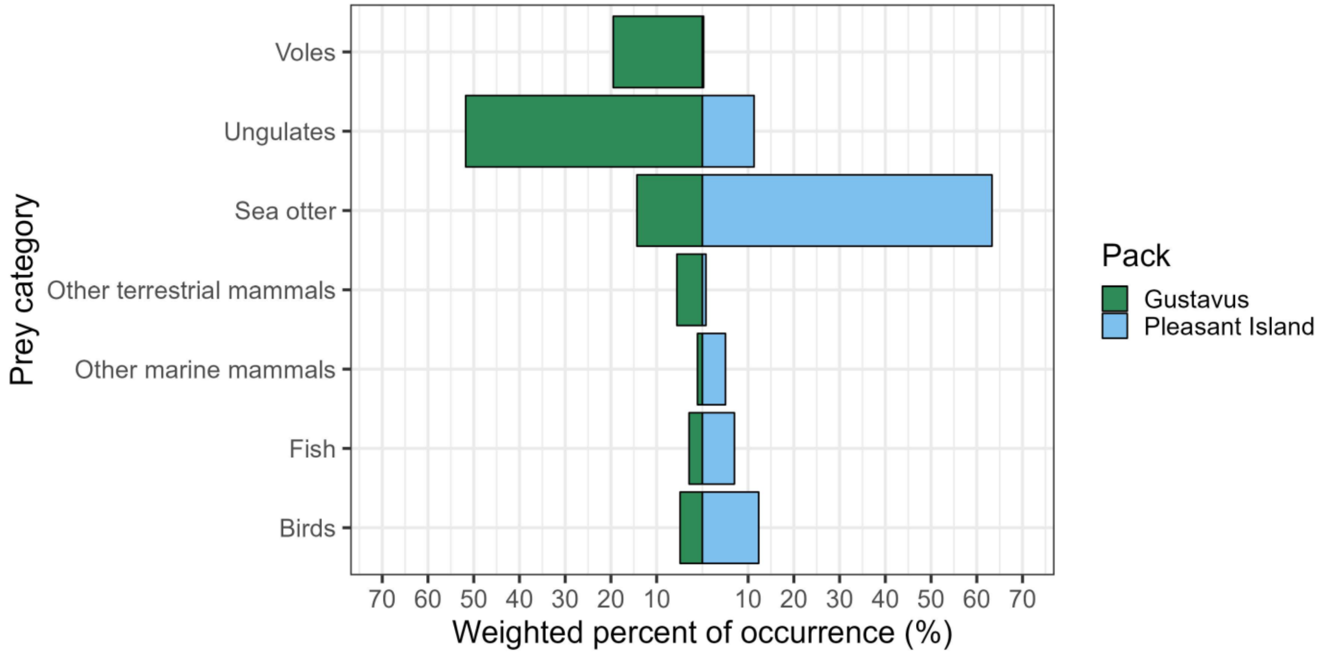

Figure S1. Dietary composition of Gustavus and Pleasant Island (Alaska, USA) wolf packs determined using 12S DNA metabarcoding of 957 scats collected from 2016-2022. Prey species were grouped into taxonomic categories and presented as weighted percent of occurrence (wPOO). For species-level diet see Table S2.

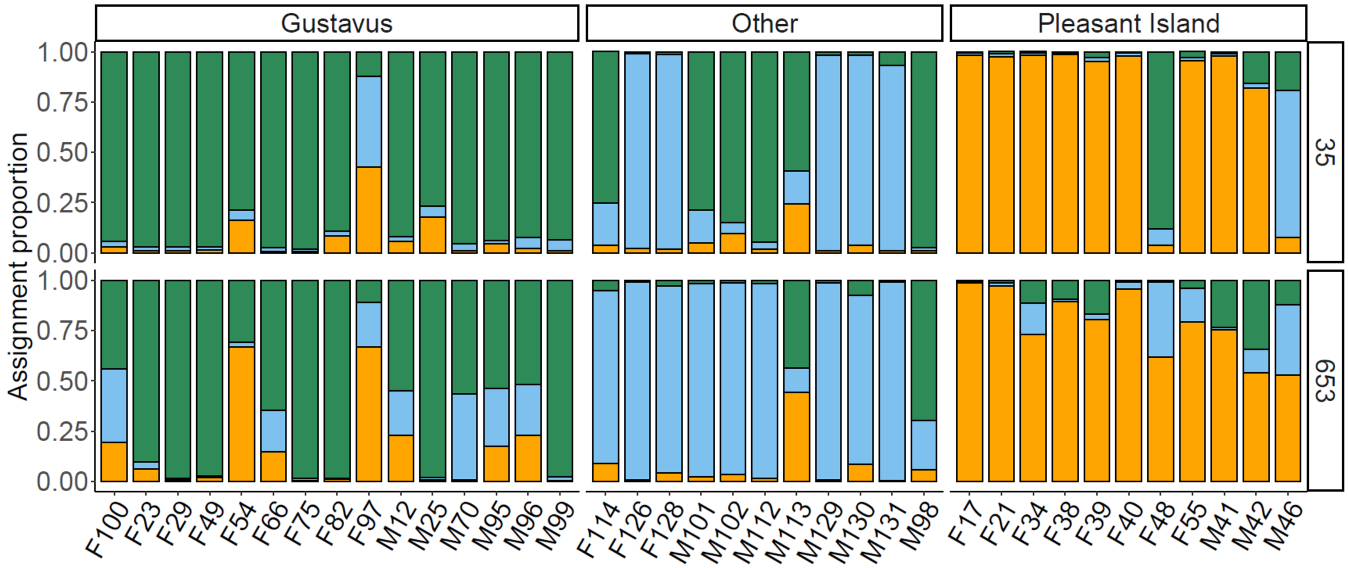

Figure S2. Comparison of proportion ancestry (pack) assignment for each wolf with tissue sample genotyped with 653 versus 35 SNPs. Each bar represents a unique genotype and the colors represent proportion ancestry to the different packs or territories. Most likely K value = 3. The regional samples that were not collected near Gustavus or on Pleasant Island have been grouped into “Other”.

Table S1. Scat samples collected per year on Pleasant Island and Gustavus Forelands in Southeast Alaska, USA.

Unit	Year	Scats collected	Other carnivore	Samples genotyped	Coyote samples
Gustavus	2016	13	0	13	4
Gustavus	2017	9	0	9	0
Gustavus	2018	46	0	44	4
Gustavus	2019	166	3	153	19
Gustavus	2020	120	0	117	31
Gustavus	2021	348	4	226	44
Gustavus	2022	89	0	0	8
Pleasant Island	2016	6	0	6	0
Pleasant Island	2017	27	0	24	0
Pleasant Island	2018	74	0	73	0
Pleasant Island	2019	56	1	52	0
Pleasant Island	2020	79	0	75	0
Pleasant Island	2021	112	0	112	0
Pleasant Island	2022	98	0	98	0

Table S2 Prey species occurrence based on DNA metabarcoding of wolf scats (n = 957) collected on Pleasant Island and the nearby Gustavus mainland from 2016 to 2022. Prey species are sorted by class and abundance.

Pleasant Island			
**Class**	**Scientific name**	**Common name**	**n scats**
Actinopterygii	*Cottidae spp.*	Sculpin spp.	19
	Pholis laeta	Crescent gunnel	13
	Hemilepidotus hemilepidotus	Red Irish lord	6
	Hexagrammos lagocephalus	Rock greenling	5
	Oncorhynchus spp.	Salmon spp.	5
	Liparis mucosus	Slimy snailfish	5
	Apodichthys fucorum	Rockweed gunnel	4
	Leptocottus armatus	Pacific staghorn sculpin	3
	Pleuronectidae spp.	Righteye flounder spp.	2
	Oncorhynchus kisutch	Coho salmon	1
	Hexagrammos decagrammus	Kelp greenling	1
	Clupea pallasii	Pacific herring	1
	Icosteus aenigmaticus	Ragfish	1
	Actinopterygii spp.	Ray-finned fish spp.	1
	Liparis gibbus	Variegated snailfish	1
Aves	*Melanitta perspicillata*	Surf scoter	17
	Anatidae spp.	Anatid spp.	14
	Haliaeetus leucocephalus	Bald eagle	11
	Turdidae spp.	Thrush spp.	9
	Melanitta deglandi	White-winged scoter	9
	Corvus spp.	Corvid spp.	6
	Gavia pacifica	Pacific loon	6
	Anas platyrhynchos	Mallard	5
	Anas americana	American wigeon	3
	Mergus spp.	Merganser spp.	3
	Bucephala clangula	Common goldeneye	2
	Dendragapus fuliginosus	Sooty grouse	2
	Larus spp.	Gull spp.	1
	Clangula hyemalis	Long-tailed duck	1
	Lagopus spp.	Ptarmigan spp.	1
	Bubo scandiacus	Snowy owl	1
	Ixoreus naevius	Varied thrush	1
	Meleagris gallopavo	Wild turkey	1
Mammalia	*Enhydra lutris*	Sea otter	304
	Odocoileus hemionus	Sitka black-tailed deer	51
	Phoca vitulina	Harbor seal	25
	Eumetopias jubatus	Steller sea lion	9

	Alces alces	Moose	8
	Lontra canadensis	North American river otter	3
	Phocoena phocoena	Harbour porpoise	2
	Myodes rutilus	Northern red-backed vole	2
	Erethizon dorsatum	North American porcupine	1

Gustavus Mainland

Class	Species	Common	n scats
Actinopterygii	*Oncorhynchus spp.*	Salmon spp.	21
	Hippoglossus stenolepis	Pacific halibut	10
	Oncorhynchus kisutch	Coho salmon	7
	Pholis laeta	Crescent gunnel	1
	Hexagrammos decagrammus	Kelp greenling	1
	Clupea pallasii	Pacific herring	1
	Ammodytes personatus	Pacific sandlance	1
	Hemilepidotus hemilepidotus	Red Irish lord	1
	Sebastes spp.	Rockfish spp.	1
	Liparis mucosus	Slimy snailfish	1
Aves	*Anatidae spp.*	Anatid spp.	13
	Corvus spp.	Corvid spp.	7
	Turdidae spp.	Thrush spp.	6
	Gallinago delicata	Wilson's snipe	6
	Haliaeetus leucocephalus	Bald eagle	4
	Meleagris gallopavo	Wild turkey	4
	Gallus gallus	Domestic chicken	2
	Larus spp.	Gull spp.	2
	Anas platyrhynchos	Mallard	2
	Melanitta perspicillata	Surf scoter	2
	Ixoreus naevius	Varied thrush	2
	Dendragapus obscurus	Dusky grouse	1
	Ardea herodias	Great blue heron	1
	Gavia pacifica	Pacific loon	1
	Pinicola enucleator	Pine grosbeak	1
	Antigone canadensis	Sandhill crane	1
	Melospiza melodia	Song sparrow	1
	Dendragapus fuliginosus	Sooty grouse	1
Mammalia	*Alces alces*	Moose	325
	Microtus longicaudus	Long-tailed vole	157
	Enhydra lutris	Sea otter	110
	Odocoileus hemionus	Sitka black-tailed deer	70
	Ursus americanus	American black bear	22
	Castor canadensis	American beaver	20

Oreamnos americanus	Mountain goat	12
Phoca vitulina	Harbor seal	11
Microtus oeconomus	Tundra vole	8
Gulo gulo	Wolverine	8
Marmota caligata	Hoary marmot	6
Myodes rutilus	Northern red-backed vole	6
Tamiasciurus hudsonicus	American red squirrel	5
Ursus arctos	Brown bear	5
Microtus spp.	Meadow vole spp.	4
Eumetopias jubatus	Steller sea lion	4
Martes americana	American marten	3
Sorex spp.	Shrew spp.	3
Erethizon dorsatum	North American porcupine	2
Ovis aries	Domestic sheep	1
Lontra canadensis	North American river otter	1

Table S3. Differences in dietary composition among individual wolves from Pleasant Island (PI) and Gustavus (GST) based on Bray–Curtis distances and permutational multivariate analysis of variance.

ID1	ID2	R2	F value	p value	Pack
F48	M25	0.146	10.754	0.002	PI/GST
F48	F29	0.364	41.771	0	PI/GST
F48	F23	0.273	24	0	PI/GST
F48	F75	0.334	33.088	0	PI/GST
F48	F82	0.128	11.348	0.001	PI/GST
F48	M80	0.091	6.489	0.012	PI/GST
F48	M12	0.226	16.388	0	PI/GST
F48	M13	0.311	24.881	0	PI/GST
F48	F49	0.301	30.943	0	PI/GST
F48	M28	0.204	17.924	0.001	PI/GST
M25	M16	0.18	15.108	0.001	GST/PI
M25	F17	0.173	19.858	0	GST/PI
M25	F21	0.248	15.791	0	GST/PI
M25	F34	0.114	4.104	0.049	GST/PI
F29	M16	0.393	51.22	0	GST/PI
F29	F17	0.384	65.398	0	GST/PI
F29	F21	0.503	58.601	0	GST/PI
F29	F34	0.319	19.679	0	GST/PI
F23	M16	0.313	31.903	0	GST/PI
F23	F17	0.297	40.527	0	GST/PI
F23	F21	0.439	38.347	0	GST/PI
F23	F34	0.265	11.919	0.002	GST/PI
F75	M16	0.363	41.054	0	GST/PI
F75	F17	0.346	51.956	0	GST/PI
F75	F21	0.487	48.407	0	GST/PI
F75	F34	0.32	16.48	0	GST/PI
F82	M16	0.17	16.973	0.001	GST/PI
F82	F17	0.174	22.883	0	GST/PI
F82	F21	0.203	15.831	0	GST/PI
F82	F34	0.078	3.907	0.051	GST/PI
M80	M16	0.127	10.32	0.003	GST/PI
M80	F17	0.127	14.071	0.001	GST/PI
M80	F21	0.178	10.85	0.003	GST/PI
M80	F34	0.05	1.789	0.212	GST/PI
M12	M16	0.245	20.153	0	GST/PI
M12	F17	0.224	25.381	0	GST/PI
M12	F21	0.421	29.788	0	GST/PI
M12	F34	0.273	9.397	0.01	GST/PI
M16	M13	0.326	29.515	0	PI/GST
M16	F49	0.337	39.594	0	PI/GST

M16	M28	0.247	24.925	0	PI/GST
M13	F17	0.295	36.398	0	GST/PI
M13	F21	0.522	43.634	0	GST/PI
M13	F34	0.408	16.557	0.001	GST/PI
F49	F17	0.329	50.953	0	GST/PI
F49	F21	0.426	42.27	0	GST/PI
F49	F34	0.249	13.617	0.001	GST/PI
M28	F17	0.247	33.525	0	GST/PI
M28	F21	0.319	25.725	0.001	GST/PI
M28	F34	0.142	6.468	0.016	GST/PI
M25	F29	0.053	2.456	0.126	GST
M25	F23	0.082	3.122	0.084	GST
M25	F75	0.051	1.98	0.18	GST
M25	F82	0.003	0.153	0.862	GST
M25	M80	0.021	0.767	0.468	GST
M25	M12	0.083	2.449	0.128	GST
M25	M13	0.201	6.536	0.012	GST
M25	F49	0.025	1.099	0.366	GST
M25	M28	0.05	2.162	0.159	GST
F29	F23	0.135	7.052	0.002	GST
F29	F75	0.005	0.217	0.855	GST
F29	F82	0.095	6.122	0.008	GST
F29	M80	0.127	6.701	0.008	GST
F29	M12	0.138	5.905	0.009	GST
F29	M13	0.276	13.709	0.001	GST
F29	F49	0.01	0.522	0.651	GST
F29	M28	0.123	7.122	0.004	GST
F23	F75	0.1	4.211	0.025	GST
F23	F82	0.107	5.876	0.01	GST
F23	M80	0.072	2.862	0.115	GST
F23	M12	0.017	0.494	0.651	GST
F23	M13	0.074	2.166	0.159	GST
F23	F49	0.117	5.846	0.009	GST
F23	M28	0.015	0.638	0.53	GST
F75	F82	0.087	4.876	0.024	GST
F75	M80	0.117	5.188	0.024	GST
F75	M12	0.122	4.154	0.023	GST
F75	M13	0.254	9.875	0.001	GST
F75	F49	0.013	0.596	0.596	GST
F75	M28	0.099	4.842	0.028	GST
F82	M80	0.02	1.034	0.376	GST
F82	M12	0.101	4.6	0.025	GST
F82	M13	0.196	9.74	0.001	GST
F82	F49	0.053	3.169	0.077	GST

F82	M28	0.075	4.452	0.031	GST
M80	M12	0.056	1.709	0.226	GST
M80	M13	0.155	5.14	0.033	GST
M80	F49	0.094	4.643	0.033	GST
M80	M28	0.028	1.258	0.309	GST
M12	M13	0.128	2.79	0.11	GST
M12	F49	0.121	4.954	0.015	GST
M12	M28	0.003	0.118	0.862	GST
M13	F49	0.249	11.625	0	GST
M13	M28	0.058	2.013	0.207	GST
F49	M28	0.108	6.032	0.012	GST
F48	M16	0.016	1.637	0.234	PI
F48	F17	0.017	2.121	0.167	PI
F48	F21	0.02	1.539	0.239	PI
F48	F34	0.016	0.966	0.376	PI
M16	F17	0.001	0.164	0.826	PI
M16	F21	0.002	0.172	0.826	PI
M16	F34	0.026	1.807	0.217	PI
F17	F21	0	0.035	0.938	PI
F17	F34	0.029	2.806	0.11	PI
F21	F34	0.056	2.726	0.126	PI
